# Short-term endothelial cell density changes after gonioscopy-assisted transluminal trabeculotomy

**DOI:** 10.5935/0004-2749.20220052

**Published:** 2025-08-21

**Authors:** Bruno Mendes de Faria, Gustavo Henrique de Lima Melillo, Fabio Daga, Fabio Nishimura Kanadani, Tiago Santos Prata

**Affiliations:** 1 Hospital Universitário Onofre Lopes, Natal, RN, Brazil; 2 Department of Glaucoma, Instituto de Olhos Ciências Médicas, Belo Horizonte, MG, Brazil; 3 Department of Ophthalmology, Universidade Federal de São Paulo, São Paulo, SP, Brazil; 4 Ver Excelência em Oftalmologia, Goiânia, GO, Brazil; 5 Department of Ophthalmology, Mayo Clinic, Jacksonville, FL, USA

**Keywords:** Glaucoma, open-angle, Corneal endothelial cell loss, Cataract extraction, Trabecular meshwork, Gonioscopy/methods, Trabeculectomy/methods, Glaucoma de ângulo aberto, Perda de células endoteliais da córnea, Extração de catarata, Malha trabecular, Gonioscopia/métodos, Trabeculectomia/métodos

## Abstract

**Purpose:**

To investigate the reduction in corneal endothelial cell density associated with gonioscopy-assisted transluminal trabeculotomy (GATT) in a short-term follow-up period.

**Methods:**

A retrospective analysis of the medical charts of patients with open-angle glaucoma who underwent gonioscopy-assisted transluminal trabeculotomy isolated or combined with phacoemulsification (phaco-gonioscopy-assisted transluminal trabeculotomy) was conducted. Patients who underwent phacoemulsification alone were included as controls. The endothelial cell density data (assessed using a specular microscope) before and at the first month after operation were collected and then compared.

**Results:**

Sixty-two eyes previously treated with gonioscopy-assisted transluminal trabeculotomy (gonioscopy-assisted transluminal trabeculotomy, n=39 eyes; phaco-gonioscopy-assisted transluminal trabeculotomy, n=23 eyes) fulfilled the inclusion criteria. The mean age of the study patients was 61.3 ± 18.4 years in the stand-alone gonioscopyassisted transluminal trabeculotomy group and 60.4 ± 11.9 in phaco-gonioscopy-assisted transluminal trabeculotomy group. Men comprised 66.6% of the patients in the isolated gonioscopyassisted transluminal trabeculotomy group and 56.5% of those in the phaco-gonioscopy-assisted transluminal trabeculotomy group. The mean visual field defects (mean deviation index) were -13.9 ± 9.2 and -10.3 ± 7.7 dB in the isolated gonioscopy-assisted and phaco-gonioscopy-assisted transluminal trabeculotomy groups, respectively. The patients in the former group presented a mean endothelial cell density reduction of 28.8 cells/mm^2^ (1.31%; p=0.467). In the latter group, the mean endothelial cell density loss was 89.4 cells/mm^2^ (4.36%; p=0.028). The control eyes (23 patients) presented a mean endothelial cell density change of 114.1 ± 159.8 cells/mm^2^ (4.41%; p=0.505). The endothelial cell density reduction in the phaco-gonioscopy-assisted transluminal trabeculotomy group was not significantly different from that in the controls (p=0.81).

**Conclusions:**

Gonioscopy-assisted transluminal trabeculotomy appears to be a safe procedure for the corneal endothelial cell layer when performed either isolated or combined with cataract extraction in a short-term follow-up period.

## Abstract (pt)

**Objetivo:**

Investigar a redução na densidade celular endotelial corneana associada à trabeculotomia transluminal assistida por gonioscopia (GATT) em curto prazo.

**Métodos:**

Análise retrospectiva de prontuários médicos de pacientes com glaucoma de ângulo aberto que foram submetidos à trabeculotomia transluminal assistida por gonioscopia isolada ou combinada com facoemulsificação. Pacientes que foram submetidos à facoemulsificação isolada foram incluídos como controles. Dados da densidade celular endotelial corneana (avaliada através de microscópio especular) pré-operatória e ao primeiro mês pós-operatório foram coletados e comparados.

**Resultados:**

Sessenta e dois olhos que foram submetidos à trabeculotomia transluminal assistida por gonioscopia (trabeculotomia transluminal assistida por gonioscopia=39 olhos; faco com trabeculotomia transluminal assistida por gonioscopia=23 olhos) passaram pelos critérios de inclusão. A idade média dos pacientes estudados era 61,3 ± 18,4 anos no grupo trabeculotomia transluminal assistida por gonioscopia isolada e 60,4 ± 11,9 anos no grupo faco com trabeculotomia transluminal assistida por gonioscopia. Homens eram 66,6% do grupo trabeculotomia transluminal assistida por gonioscopia isolada e 56,5% do grupo faco com trabeculotomia transluminal assistida por gonioscopia. O defeito perimétrico médio (*Mean Deviation*) era -13,9 ± 9,2 dB e -10,3 ± 7,7 dB nos grupos trabeculotomia transluminal assistida por gonioscopia isolada e faco com trabeculotomia transluminal assistida por gonioscopia respectivamente. O grupo que fora submetido à trabeculotomia transluminal assistida por gonioscopia isolada apresentou redução média da densidade celular endotelial corneana de 28,8 células/mm^2^ (1,31%; p=0,467). No grupo faco com trabeculotomia transluminal assistida por gonioscopia, a redução média da densidade celular endotelial corneana foi de 89,4 células/mm^2^ (4,36%; p=0,028). Olhos controle (23 olhos) apresentaram redução média da densidade celular endotelial corneana de 114,1 ± 159,8 células/mm^2^ (4,41%; p=0,505). A redução na densidade celular endotelial corneana no grupo faco com trabeculotomia transluminal assistida por gonioscopia não foi significativamente diferente do grupo controle (p=0,81).

**Conclusões:**

A trabeculotomia transluminal assistida por gonioscopia parece ser segura para a camada endotelial corneana em um curto prazo quando realizada de forma isolada ou combinada com cirurgia de catarata.

## INTRODUCTION

Glaucoma is a multifactorial optic neuropathy characterized by the slow, progressive degeneration of retinal ganglion cells and axon loss^(1)^. Although its pathophysiology is not fully understood, elevated intraocu lar pressure (IOP) is strongly related to the development and progression of the disease^(1-3)^. Consequently, treatment relies on IOP reduction as an approach to prevent further structural and functional damages^(1-3)^. For decades, the traditional options to reduce IOP included topical hypotensive medications, laser therapy (trabeculoplasty), and conventional incisional (filtering) procedures. More recently, different procedures based on the concept of microinvasive glaucoma surgery (MIGS) have emerged as faster and safer alternatives for cases of open-angle glaucoma for which moderate IOP reductions are intended^(4,5)^.

Ab interno trabeculotomy known as gonioscopyassisted transluminal trabeculotomy (GATT) is a technique described by Grover et al. as an innovative surgical approach for open-angle^(6)^, primary congenital, and juvenile-onset glaucomas^(7)^. In brief, GATT reduces the IOP by improving the flow of aqueous through Schlemm’s canal, keeping intact the conjunctival tissue; hence, it is considered a MIGS procedure. Overall, good outcomes regarding IOP control and reduction in glaucoma medication use have been reported^(6,8)^.

Regarding the safety profile of GATT, associated serious or sight-threatening postoperative complications are uncommon^(6,8)^. The most common complications include transitory hyphema, prolonged ocular inflammation, and steroid induced IOP increase. Unwanted postoperative findings related to intraoperative surgical complications such as iridodialysis, cyclodialysis cleft, and Descemet detachment have been scarcely reported^(8)^. The contraindications of GATT include bleeding diathesis, anticoagulation, closed angle, and severe endothelial dysfunction^(6)^. No possible effects on corneal endothelial cell density (ECD), which is a major concern in all intraocular surgeries (no less important in MIGS^(9)^), have been reported to date. Differently from most MIGS procedures, GATT does not involve the implantation of a microstent or tube in the anterior chamber, but the surgical manipulation itself could potentially affect ECD nonetheless. Therefore, we aimed to investigate shortterm ECD changes associated with GATT as an isolated procedure or combined with phacoemulsification in open-angle glaucoma eyes.

## METHODS

This retrospective, interventional case series study adhered to the tenets of the Declaration of Helsinki and to the institutional review board guidelines of Federal University of Rio Grande do Norte.

### Participants and data collection

All the medical charts of glaucomatous patients who underwent GATT either as an isolated procedure or combined with phacoemulsification (phaco-GATT) between January 2017 and February 2020 were reviewed. All the patients who underwent GATT who had uncontrolled IOP were receiving maximally tolerated medical therapy. To be included in the study, patients must have complete information about the procedure (based on each patient’s surgical report) and detailed preoperative and postoperative data, including IOP and endothelial cell count measurements. Eyes that presented with any corneal disease or ocular trauma, or that had been treated with any intraocular surgery or laser procedure within 6 months before the GATT procedure were excluded.

The data collected included preoperative and postoperative IOPs, number of antiglaucoma medications, ECD values (based on automated specular microscopic examination results), surgical complications, and any subsequent related events or procedures. As the focus of this study was to assess the short-term ECD changes directly related to the surgical procedure (GATT) itself and as GATT does not involve the implantation of any device in the anterior chamber (e.g., a microstent or tube), postoperative outcomes were evaluated at 1 month after surgery. To better investigate the ECD changes in the phaco-GATT group, a separate group of non-glaucomatous patients who had previously undergone isolated cataract extraction (phacoemulsification) were included as controls for comparison. The main outcomes were ECD changes after GATT and phaco-GATT. As a secondary outcome, we investigated the profile of the patients with significant ECD loss, defined as a reduction >20% (based on the 95th percentile of the ECD change distribution) as compared with the baseline measurement.

### Surgical procedure

All the surgeries were standardized and performed by one of the authors (B.M.F.) at a single center (Onofre Lopes University Hospital). Briefly, after superior nasal and temporal corneal paracentesis, a solution containing lidocaine and carbachol was injected in the anterior chamber. Afterwards, the anterior chamber was filled with a viscoelastic substance (methylcellulose 2%). By using the tip of a 26-gauge needle, a nasal goniotomy was created. A thermally blunted 5-0 polypropylene suture was then inserted through the goniotomy and circumferentially advanced with the aid of a 23-gauge serrated-tip microsurgical forceps. The distal tip of the suture was advanced by 360º and was retrieved at the nasal goniotomy site and extracted from the anterior chamber, creating a circumferential trabeculotomy. In some cases where anatomical resistance was detected while advancing the suture, a new goniotomy was created to retrieve the distal tip, creating a circumferential trabeculotomy that ranged from 90° up to 360°. The viscoelastic substance was then removed from the anterior chamber by anterior chamber irrigation with a basic saline solution. Acute hypotony and hyphema were controlled using an anterior chamber viscoelastic injection. The amount of viscoelastic substance left in the eye was determined on the basis of the degree of blood reflux and presence and degree of episcleral venous fluid wave.

In patients for whom GATT was combined with cataract extraction (phaco-GATT group), phacoemulsification with intraocular lens implantation was performed first. Then, the trabecular meshwork was accessed with nasal goniotomy after the ab interno trabeculotomy. Intraoperative intracameral carbachol was used at the end of the phacoemulsification, before initiating the GATT procedure. The same paracenthesis was used for goniotomy and phacoemulsification, without any difference from the ordinary phacoemulsification surgical technique. All the patients were treated postoperatively with topical moxifloxacin (4 times daily for a weeks), pilocarpine 2% (2 times daily for 2 weeks), and corticosteroid (prednisolone 1%). The topical corticosteroids were initially applied every 2 hours, and this dose was then gradually tapered over the first month of treatment.

### Statistical analysis

Descriptive analysis was used to obtain demographic and clinical data. The D’Agostino-Pearson test was performed to determine whether the data had a normal distribution. The normally distributed data were presented as mean and standard deviation, whereas the nonnormally distributed data were presented as median and interquartile intervals. Between the preoperative and postoperative parameters, continuous data were compared using the paired-samples *t* test or Wilcoxon signed-rank test, depending on the data distribution. An independent samples *t* test was used to compare the magnitude of ECD changes between the eyes treated with phaco-GATT and the controls (isolated cataract extraction). Computerized analysis was performed using the MedCalc software (MedCalc Inc., Mariakerke, Belgium), and statistical significance was set at p<0.05.

## RESULTS

We reviewed the charts of 90 patients who had previously undergone the GATT procedure. Among the patients, 62 (62 eyes) fulfilled our inclusion criteria (GATT, n=39 eyes; phaco-GATT, n=23 eyes). Detailed information about their demographics and ocular characteristics are provided on table 1. Among the patients diagnosed as having secondary open-angle glaucoma, 10 had cortisone glaucoma, 2 had congenital glaucoma, and 2 had pigmentary glaucoma.

**Table 1:** Baseline demographic and ocular characteristics of the study patients

Variable	Patients	
	GATT (n=39)	Phaco-GATT (n=23)
Age (years)^*^	61.3 ± 18.4	**60.4 ±11.9**
Sex (men/women)	26/13	13/10
Diagnosis (POAG/SOAG)	28/11	20/3
VFMD index (dB)^*^	-13.9 ± 9.2	-10.3 ± 7.7
Number of previous intraocular surgeries†	1 (1,1)	0(0,1)
Preoperative intraocular pressure (mmHg)^*^	24.0 ± 7.8	27.0 ± 8.3
Number of preoperative medications^*^	3.6 ± 0.5	3.5 ± 0.5
Preoperative ECD (cells/mm^2^)^*^	2181.6 ± 481.2	2136.9 ± 418.6

POAG= primary open-angle glaucoma; SOAG= secondary open-angle glaucoma; VFMD= visual field mean deviation; ECD= endothelial cell density.

* Data are presented as mean ± standard deviation.

† Non-normally distributed variables represented by median (first quartile, third quartile).

**Figure 1:**
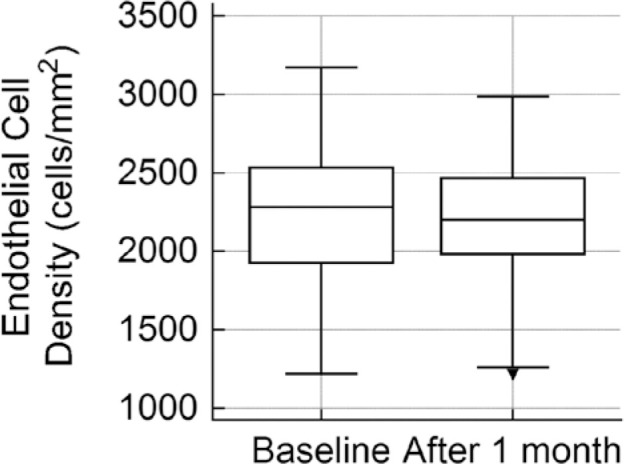
Box-and-whisker plot depicting endothelial cell densities before and after the isolated GATT procedure.

**Figure 2:**
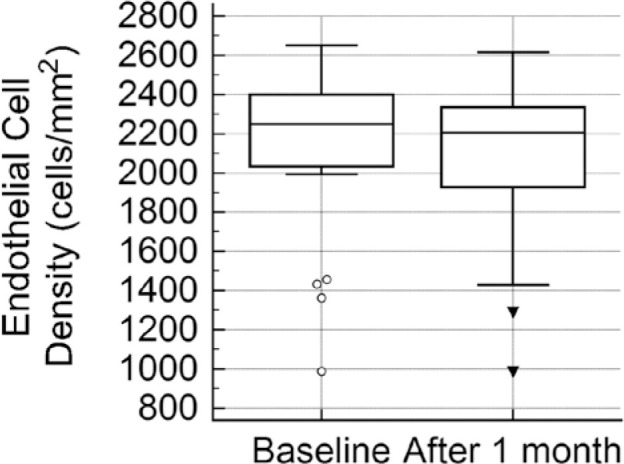
Box-and-whisker plot depicting endothelial cell densities before and after the combined GATT and cataract surgery.

Regarding the ECD analyses, the isolated GATT group had a mean preoperative ECD of 2181.6 ± 481.2 cells/ mm^2^. On the 30th postoperative day, the mean ECD was 2152.8 ± 425.39 cells/mm^2^ (Figure 1). The mean difference of 28.8 cells/mm^2^ (1.31%) was not significant (p=0.467). In the phaco-GATT group (Figure 2), the mean preoperative ECD was significantly reduced from 2136.9 ± 418.6 cells/mm^2^ to 2047.5 ± 421.6 cells/mm^2^ (mean difference, 89.4 cells/mm^2^ [4.36%; p=0.028]). When comparing the ECD changes documented for the control eyes (mean ECD change, 114.1 ± 159.8 cells/ mm^2^ [4.41%]; p=0,505), which had been treated with isolated cataract extraction (23 patients; mean age, 67.5 ± 9.1 years), we found no significant difference (p=0.815).

Overall, the mean IOP was significantly reduced on the 30th postoperative day (from 24.0 ± 7.8 mmHg preoperatively to 11.0 ± 2.1 mmHg postoperatively in the isolated GATT group [p<0.001] and from 27.0 ± 8.3 mmHg preoperatively to 12.4 ± 4.0 mmHg postoperatively in the phaco-GATT group [p<0.001]). On the 30th postoperative day, the mean number of antiglaucoma medications was significantly reduced from 3.6 ± 0.5 to 0.8 ± 1.0 in the isolated GATT group (p<0.001) and from 3.5 ± 0.5 to 0.6 ± 0.9 in the phaco-GATT group (p<0.001). In the stand-alone procedure group, GATT was performed in quadrants 4, 3, 2, and 1 in 76.9%, 7.6%, 12.8%, and 2.5% of the cases, respectively. In the phaco-GATT group, 65.2%, 4.3%, 30.4%, and 0% of the eyes were treated with GATT in quadrants 4, 3, 2, and 1, respectively. No intraoperative complications (besides GATT-induced hyphema) were recorded in both groups. Postoperatively, transitory hyphema was observed in 58.9% and 43.4% of the eyes in the isolated GATT and phaco-GATT groups, respectively, with a median duration of 3 days in both groups.

Finally, regarding the subanalysis of the cases with a more pronounced ECD loss, we found four eyes with a reduction >20% as compared with the baseline measurement. Among the patients, two underwent phaco- GATT and two underwent isolated GATT. In three of the patients, GATT was performed in 360°, while in one eye, only two quadrants were treated. All the eyes had a primary open-angle glaucoma, with a mean visual field mean deviation index of -9.2 ± 8.7dB. Preoperatively, the mean IOP, ECD, and number of antiglaucoma medications were 19.7 ± 8.3 mmHg, 2301 ± 264.4 cells/ mm^2^, and 3.5 ± 0.5, respectively. On average, four eyes had previously undergone a median of 0.5 intraocular surgeries. Postoperatively, hyphema resolved within a median of 7 days in the four eyes, and no other complication was recorded. Although not based on inferential analysis, the profiles of the eyes that presented a more pronounced ECD loss after GATT seemed comparable with those of the entire study population (except for the mean baseline IOP, which was approximately 5 mmHg lower in these four eyes).

## DISCUSSION

Surgical innovation, a major drive of improvements in medical practice, plays a remarkable role in ophthalmology. New surgical techniques should not only succeed in providing an alternative approach to obtain the same or better outcomes than the conventional technique but also offer better safety regarding adverse effects^(10)^. Considering the course of glaucoma treatment, glaucomatous eyes are well known to be prone to present an increased loss of endothelial cells due to different factors (aging, clinical treatment, laser exposure, and glaucoma itself) over time^(11-14)^, which make awareness of the effect of a surgical procedure on ECD even more important. Even though previous studies disclosed GATT-related complications^(6-8)^, its possible effects on ECD have not been reported to date. By investigating short-term ECD changes in patients with open-angle glaucoma treated with GATT (with or without phacoemulsification), we found no significant changes in the average ECD. In addition, only a small percentage (6.5%) of study eyes had a more pronounced ECD loss.

Data on ECD changes after the MIGS procedure are scant in the literature, and the impact of each alternative seems to be diverse. The 5-year follow-up after supraciliary micro-stent implantation (CyPass; Alcon Laboratories Inc, Ft. Worth, TX) revealed a continued endothelial damage likely stemming from its position within the angle^(9)^. Such perception led to voluntary market withdrawal of the stent by the manufacturer. By contrast, the use of the ab interno implanted trabecular micro-bypass system (iStent; Glaukos Corporation, San Clemente, CA) resulted in no significant endothelial cell loss as compared with that in the control group in at 2-year follow-up^(2)^. The Hydrus microstent (Hydrus, Ivantis, Irvine, CA) and Xen gel stent (Allergan, Dublin, CA) did not seem to increase the ECD loss in at 6-month and 2-year follow-ups, respectively^(15,16)^. More robust evidence of ECD changes after the surgical approach for glaucoma was found in regard to filtering procedures. Overall, mitomycin-C-augmented trabeculectomy is associated with an ECD reduction of 3%-11.4% (at follow-up periods ranging from 3-24 months)^(17-22)^. Bleb needling with adjunctive use of mitomycin-C is not associated with a significant reduction in ECD^(23)^. Ex-Press shunt implantation (Alcon Laboratories Inc, Ft. Worth, TX), especially if more anteriorly placed, may significantly decrease the ECD (mean loss of 15.1% in 2 years)^(24)^. Finally, glaucoma drainage device implantation seems to expose the endothelium to a higher risk of continuous cell depletion remarkably in cases of corneal touch^(22,25,26)^. Although no comparisons with other surgical alternatives were made in the present study, our findings suggest that surgery-related (short-term) ECD changes after GATT are not clinically significant and are comparable with those observed after the use the safest MIGS available.

At this point, it is important to discuss the main clinical implications of our findings. Many glaucomatous eyes may require multiple procedures during the patients’ lifetimes. As human endothelial cells have a limited division potential^(14)^, their preservation is a major concern during intraocular surgery. Even though neither microstents nor tubes are implanted in the anterior chamber during the GATT procedure, the surgical manipulation itself could eventually damage the endothelial cells. However, in this case series, we found no significant changes after the stand-alone procedure, and the ECD loss resulting from the combined procedure was not significantly different from that observed after the isolated cataract surgery. In fact, we observed a slightly larger endothelial cell reduction in the controls than in the phaco-GATT group, which may be due to the higher mean baseline ECD of the enrolled controls. This favorable safety profile can be possibly explained by certain characteristics of the procedure as follows: use of a viscoelastic substance to depress the peripheral iris and shield endothelial cells^(27)^, gentle and precise movements in the anterior chamber, brief operative time, and low incidence rates of intraoperative and postoperative complications^(8)^. Bringing this safety information to a more clinical perspective, physicians often deal with patients with poor corneal endothelial conditions at the time of glaucoma surgery indication. Moreover, as discussed earlier, each glaucoma procedure may cause a different impact on corneal endothelial cells. In this context, we believe that awareness of surgery-related ECD changes becomes essential, allowing surgeons to choose the best option for each patient, considering both safety and efficacy outcomes.

This study has some specific characteristics and limitations that should be considered while interpreting its findings. First, although the status of the corneal endothelium is often evaluated using a quantitative parameter (ECD; cells/mm^2^), morphological changes could also be observed after surgical trauma^(11,13)^. In our study, qualitative analyses of cell size and shape were not performed. Second, as previously stated, the focus of this study was to assess the short-term ECD changes directly related to the surgical procedure. To mitigate the influence of possible confounding factors, ECD changes were evaluated at 1 month after operation. Therefore, our findings do not reflect the GATT-related ECD changes occurring over longer periods. However, as no device remains in contact with the anterior chamber over time, we believe that any additional, more pronounced ECD loss would be improbable. Third, in some cases, higher baseline IOP values and IOP changes of greater magnitude may have influenced the ECD measurements (e.g., due to corneal edema). Although our analyses did not take this covariate into consideration, we still found counterintuitive postoperative ECD changes (a paradoxical increase) in a few subjects. Fourth, the relatively small sample size of our study might have negatively impacted the statistical power of our analyses. Finally, although short-term efficacy results were reported in the present study (IOP and medications outcomes), they were only described herein for a better interpretation of the ECD outcomes. They were derived from only 30 days of follow-up and have no clinical significance for success rate analysis.

In conclusion, on the basis of the investigation of postoperative (short-term) ECD changes in a population mostly comprised of eyes with advanced primary openangle glaucoma, GATT (with or without phacoemulsification) does not lead to a clinically significant damage to corneal endothelial cells. We believe that these findings are a significant addition to the existing knowledge on the safety of MIGS and will help surgeons customize surgical options for each patient. Further longitudinal studies are warranted to confirm our findings and to investigate long-term ECD changes after GATT.
